# Trends of reported human brucellosis cases in mainland China from 2007 to 2017: an exponential smoothing time series analysis

**DOI:** 10.1186/s12199-018-0712-5

**Published:** 2018-06-19

**Authors:** Peng Guan, Wei Wu, Desheng Huang

**Affiliations:** 1 Department of Epidemiology, School of Public Health, China Medical University, Shenyang, 110122 China; 2 Department of Mathematics, School of Fundamental Sciences, China Medical University, Shenyang, 110122 China

**Keywords:** Human brucellosis, Time series analysis, Exponential smoothing method

## Abstract

**Background:**

The main objective of this study was to describe the temporal distribution of monthly reported human brucellosis cases in mainland China and develop an appropriate time series model for short-term extrapolation forecast.

**Methods:**

Surveillance data of the monthly reported human brucellosis cases occurring from April 1, 2007, to March 31, 2017, in mainland China were obtained. The spectrum analysis was first adopted to find the cyclic and seasonal features, the existence of the seasonality and trend was determined by exponential smoothing method and the seasonal-trend decomposition. The candidate models of exponential smoothing included the additive model and multiplicative model; *R*^2^ was selected as the indicator for the selection of candidate model, and the stability of the model was verified by adjusting the training data and test data set. Finally, the extrapolations of monthly incident human brucellosis cases in 2017 were made.

**Results:**

From April 1, 2007, to March 31, 2017, a total of 435,108 cases of Brucellosis occurred in mainland China were reported, with an average of 3626 cases per month and a standard deviation of 1834 cases. The *R*^2^ of the exponential smoothing method that based on additive model increased steadily from 0.927 to 0.949 with the increase of the data volume. Ten of 12 actual values fell in the confidence interval of predicted value.

**Conclusions:**

Human brucellosis cases peaked during the months from March to August in mainland China, with clear seasonality. The exponential smoothing based on the additive model method could be effectively used in the time series analysis of human brucellosis in China. Control methods, such as vaccination, quarantine, elimination of infected animals, and good hygiene within the production cycle, should be strengthened with paying more attention to the seasonality. Further research is warranted to explore the drivers behind the seasonality.

## Background

The transmission of infectious diseases between domestic and wild animals is posing great challenges to public health [1–3]. Among the numerous emerging infectious diseases, including zoonoses, human brucellosis is one of the most common zoonotic diseases. Although there has been a noticeable decrease in the incidence and prevalence of brucellosis in many countries due to their domestic animal brucellosis control or eradication programs, human brucellosis is a growing public health concern in China with the increasing number of reported cases and more widespread natural foci [4]. In mainland China, human brucellosis was first reported in 1905, the incidence of human brucellosis in China was quite severe before the 1980s and declined later [4, 5]. Comprehensive prevention and control measures have been gradually implemented in mainland China since 1950 [5]. Nationwide reporting of human brucellosis was established during 1950–1963, during 1964–1976, vaccination for animals and humans was applied in the high-risk areas, and then during 1977–1988, a national brucellosis control program was administered with the introduction of clinical features, case definition, laboratory examination criteria, treatment options, and control measures; the vaccination of livestock was selected as the main control method. Since 1990, sentinel surveillance of the seroprevalence of brucellosis in humans and animals has been added to the national brucellosis control program [6]. However, the incidence of human brucellosis has been increasing greatly since 1990, against the background that the incidence of animal brucellosis remained unchanged [7]. Also, the geographic distribution of affected regions gradually expanded, from traditional pasturing areas to agricultural areas, from the north part to the south part [4]. These kinds of variation could be attributed to various kinds of risk factors. The number of livestock increased significantly in the past decade to meet the growing demands for meat consumption in mainland China [8], which would give rise to the increase of the total population of infected domestic animals, even with relatively low-level seroprevalence in livestock. Lack of quarantine or pasteurization in the livestock product supply also increased the risk of infections in non-occupational populations and urban settings [9, 10].

Multiple publications have been dealing with the epidemiological characteristics and spatio-temporal distribution of human brucellosis [11, 12], while the studies focusing on the forecasting models were limited. The epidemic of human brucellosis is associated with the variation features of periodicity and seasonality; the major part of cases can be seen during the peak period, while sporadic cases can be observed during the non-peak period. Early recognition of the epidemics may provide strong support for the prevention and control of human brucellosis. The short-term forecast can evaluate the prevention or control measures; meanwhile, it can adopt timely and effective countermeasures for the epidemic peak that may occur. Among the available prediction models, time series analysis [13] was the most commonly adopted method, and some characteristics could be found from the annual data.

In the present study, the reported number of human brucellosis cases on a monthly basis in mainland China from 2007 to 2017 was collected and the short-term forecast was made with the seasonal-trend decomposition (STL) and exponential smoothing model.

## Methods

### Design and ethical considerations

The current study utilized a longitudinal, non-experimental design. This study was conducted on the basis of the nationwide surveillance of human brucellosis in mainland China and was exempt from institutional review board assessment. All the data in the present study were publicly open and were supplied and analyzed in an anonymous format, without access to any personal identifying information.

### Data source

The monthly reported numbers of human brucellosis cases (clinically diagnosed and laboratory confirmed) occurring from April 1, 2007, to March 31, 2017, in mainland China were obtained from the National Health Commission of the People’s Republic of China and the CDC of the People’s Republic of China. In China, the Law on the Prevention and Control of Infectious Diseases [14] requires health-care staff to report any of the 39 infectious diseases to the Center for Disease Control and Prevention (CDC) through the National Noticeable Infectious Disease Reporting system (NIDR). Human brucellosis is listed as a Class II legally mandated notifiable disease and brucellosis is listed as a Class II key disease by the Disease Prevention and Control of Livestock and Poultry in China [15].

The clinical diagnosis of human brucellosis cases was made according to the clinical manifestations (undulant fever, sweats, fatigue, myalgia, arthralgia, arthritis, hepatomegaly, splenomegaly, epididymo-orchitis, etc.) and confirmed by serologic test or isolation of the organism, in accordance with the case definition of the World Health Organization [16].

### Statistical analysis

The monthly reported number of human brucellosis cases was exported to Microsoft Excel 2016 for the distribution description. The spectrum analysis was first adopted to find the cyclic and seasonal features; the existence of the seasonality and trend was determined by exponential smoothing method and the seasonal-trend decomposition. The decomposition model assumes that the data has the following form: Times series = Pattern + Error = Trend cycle + Seasonality + Error. The candidate models of exponential smoothing included additive model and multiplicative model; *R*^2^ was selected as the indicator for the selection of candidate model, and the stability of the model was verified by adjusting the training data and test data set. Finally, the extrapolations of monthly incident human brucellosis cases in 2017 were made.

All statistical analyses were conducted by using the software IBM SPSS Statistics 21.0 for Windows (IBM, Asian Analytics Shanghai).

## Results

### Reported cases of human brucellosis cases in China

**Fig. 1:**
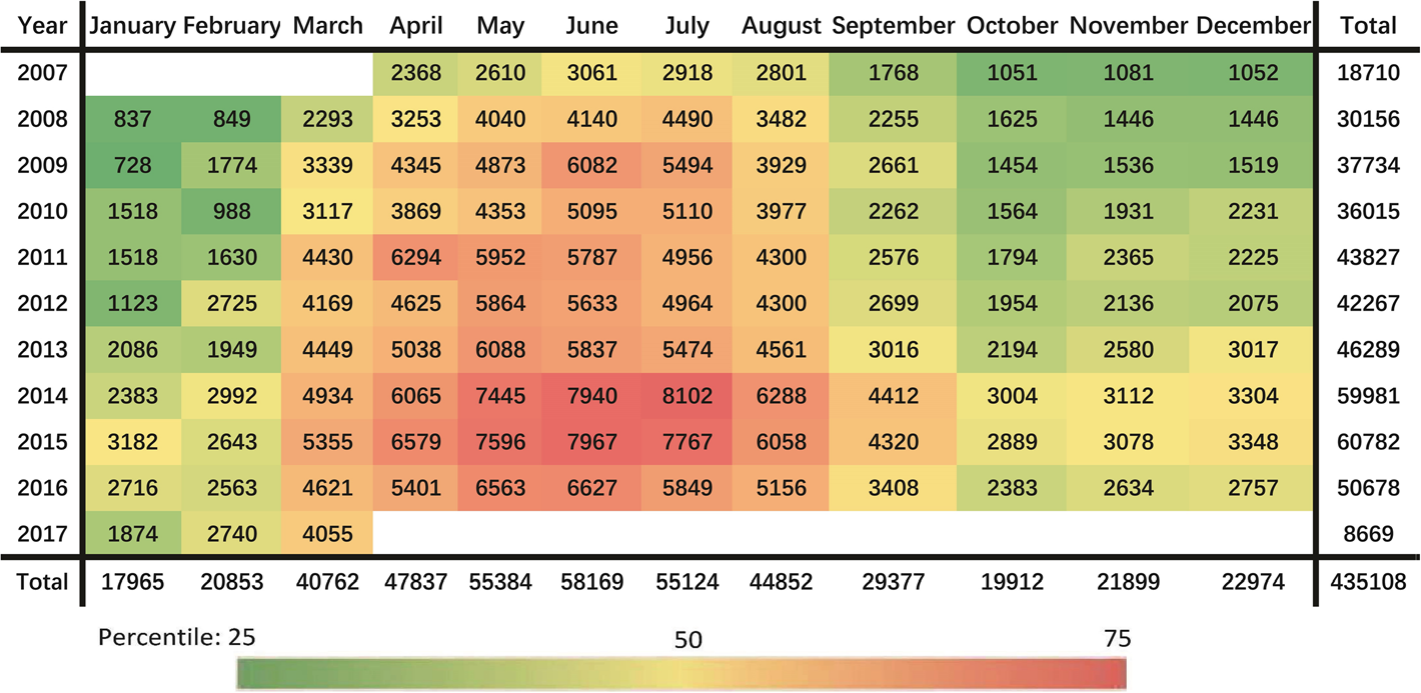
Reported cases of human brucellosis, by month of the year, in Mainland China, April 2007–March 2017

From April 1, 2007, to March 31, 2017, a total of 435,108 cases of brucellosis occurred in mainland China were reported, with an average of 3626 cases per month and a standard deviation of 1834 cases. A heat map of the monthly cases was created to visualize the long term of reported cases during the 10 years (Fig. 1). The annual increase of the reported cases is 7.8%. The peak of monthly reported cases was 8102 cases, which appeared in July 2014; the bottom was 728 cases, which appeared in January 2009.

### Seasonal features of reported brucellosis cases

Brucellosis cases peaked during the months from March to August, as observed in nearly all of the years of the study, while the most noticeable annual increase was observed from 2014 to 2015. There was a clear seasonality of monthly human brucellosis cases during the study period. During the peak season, a total of 302,128 cases were found, accounting for 69.44% of the total cases, with an average of 5035 cases and a standard deviation of 1440 cases. Overall, June was the month with the highest number of reported cases; there were 435,108 cases reported across the study period, where the peak of reported cases (60,782 cases) was in 2015. During the peak season, the peak of monthly reported cases was 8102 cases, which appeared in July 2014; the bottom was 2293 cases, which appeared in March 2008. By contrast, January had the lowest number of reported cases (17,965 cases) across the period, with the lowest monthly number (728 cases) in 2009.

**Fig. 2:**
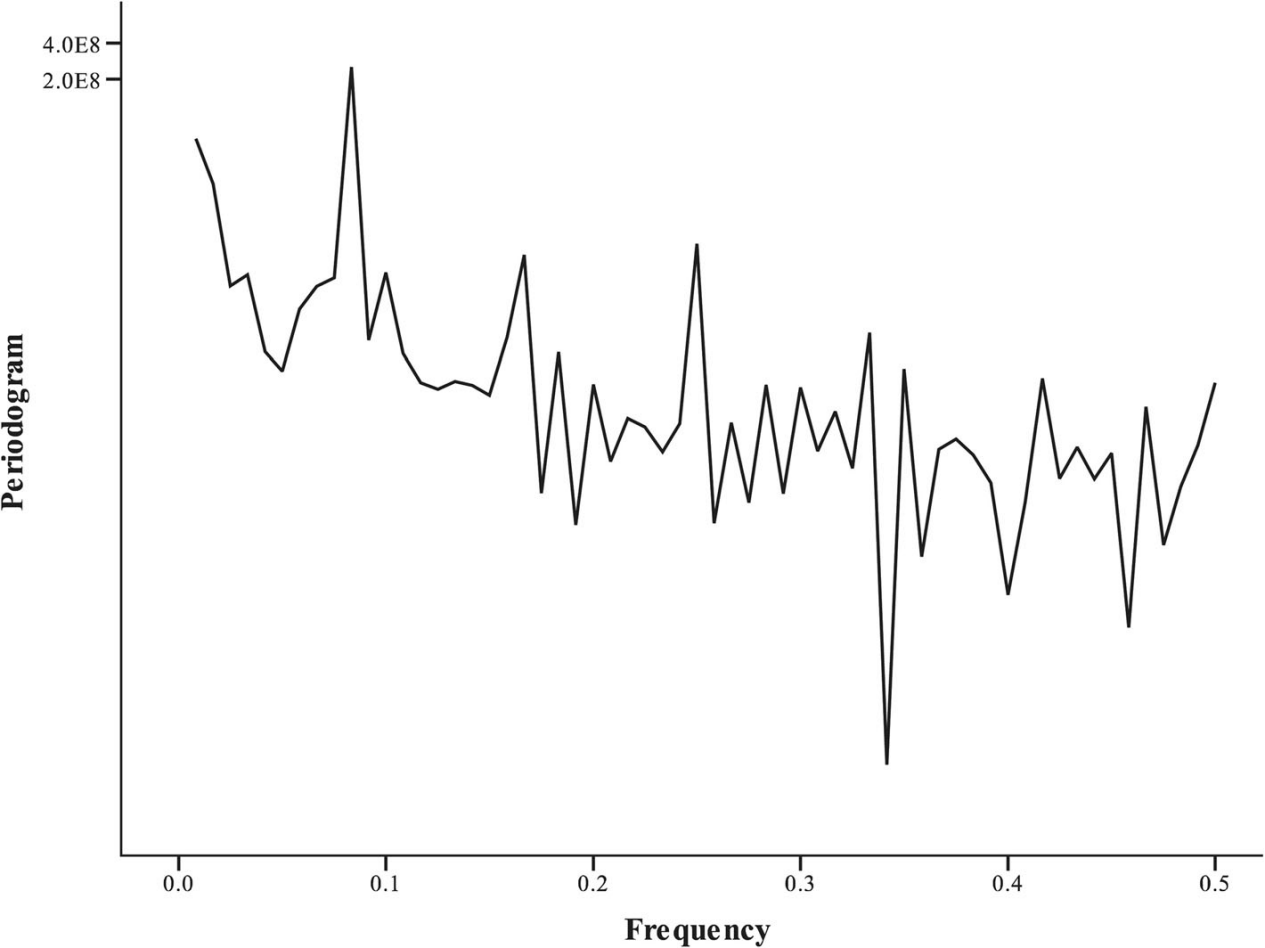
Spectrum analysis of monthly reported cases of human brucellosis in Mainland China, April 2007–March 2017

According to spectrum analysis, the frequency ◊ period = 1; it is decomposed into many sine waves by the Fourier series. From Fig. 2, it can be seen that among these waves, the amplitude of the wave with the frequency of 0.0833 is the strongest, and its reciprocal value (1/0.0833) is the period of 12 months. Taking into account the peak time of reported cases, the seasonality of human brucellosis cases was thus determined.

### Trend and periodicity of reported brucellosis cases

**Fig. 3:**
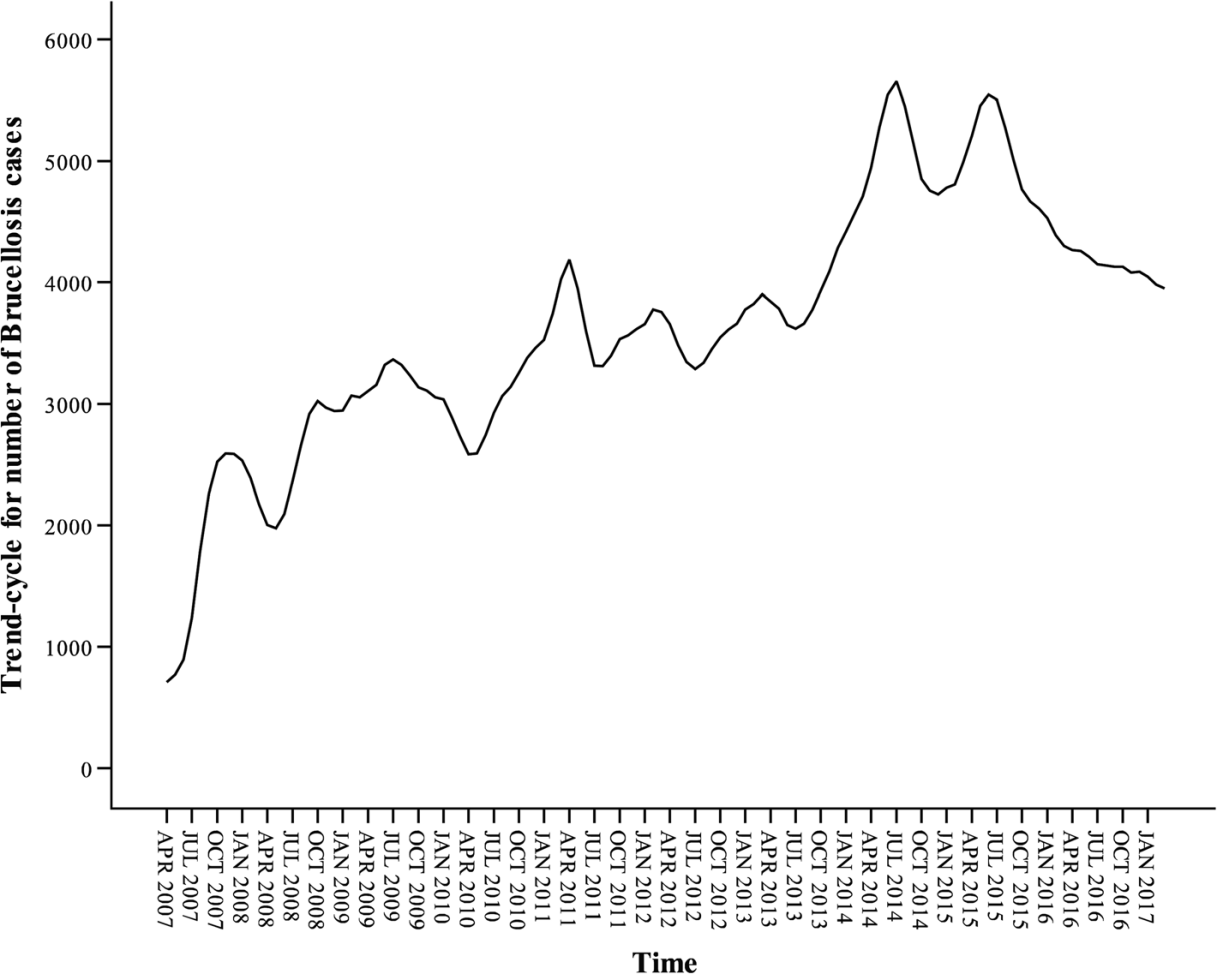
Cycle and trend of the monthly reported cases of human brucellosis in Mainland China, April 2007–March 2017 (Exponential smoothing method based on the additive model)

The seasonal decomposition of additive model indicated that there was a clear trend of increase in the monthly number of human brucellosis cases and there was an obvious upward trend from April 2007 to July 2015 (Fig. 3).

**Fig. 4:**
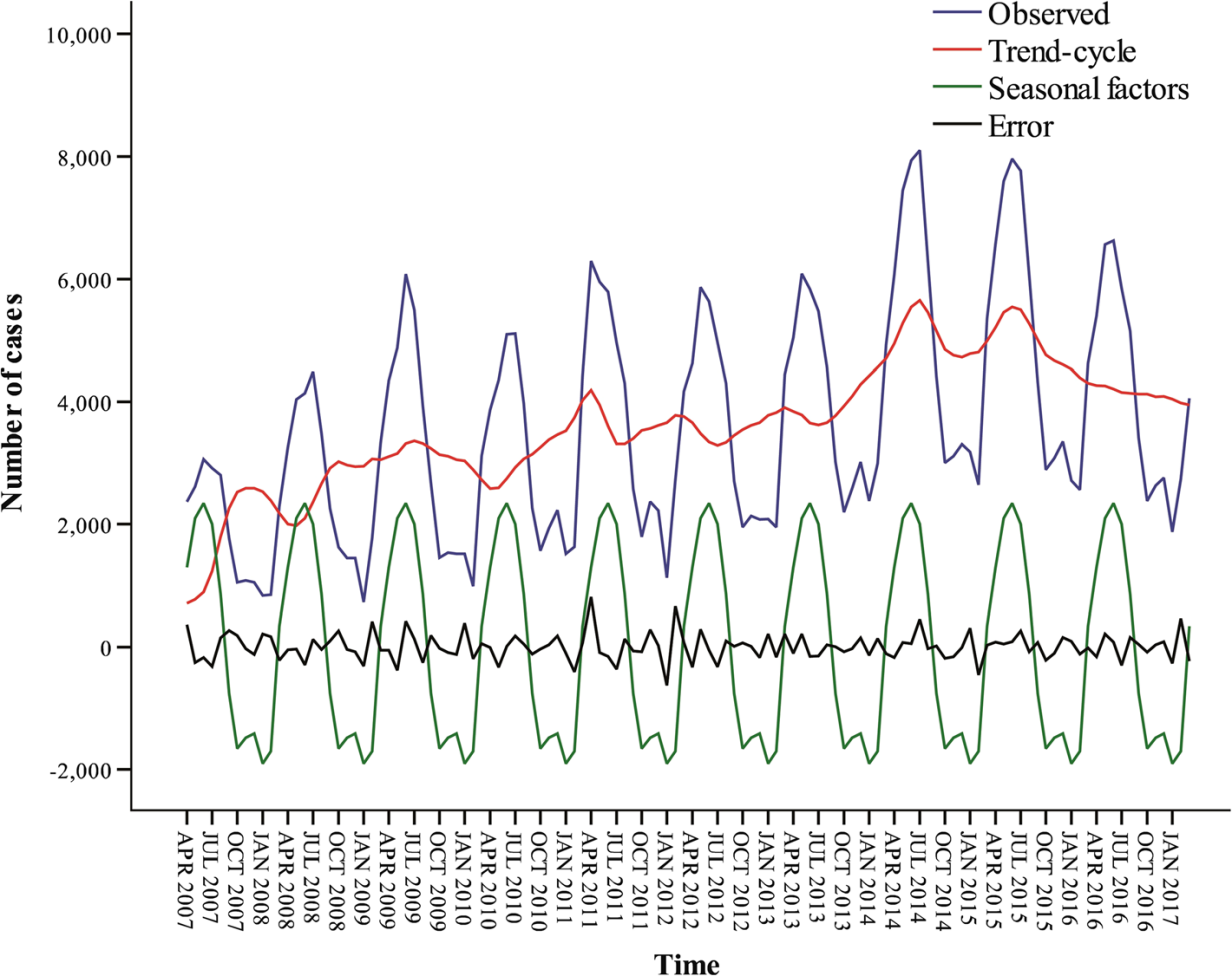
Error line between observed and fitted values in the additive model

From Fig. 3, it can be seen that in the additive model, the first cycle is from April 2008 to April 2010, the second cycle is from April 2010 to July 2012, the third cycle is from July 2012 to April 2015, and the last cycle has been ongoing since April 2015. The error between observed and fitted values in additive model ranged from − 628 to 811 (Fig. 4).

### Additive model sensitivity and forecasting performance

**Fig. 5:**
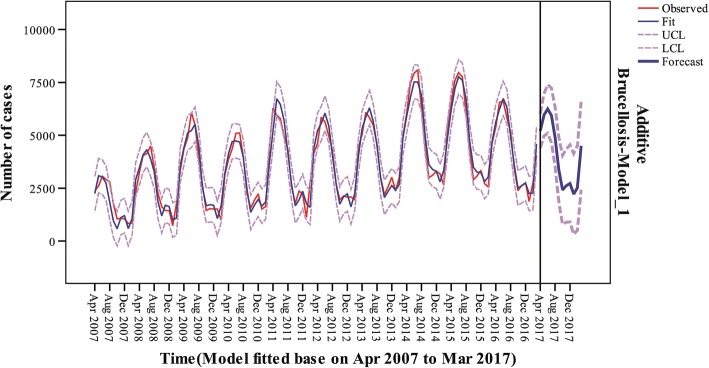
Extrapolation prediction of the monthly reported cases between April 2017 to March 2018 in Mainland China, based on the additive model

A good model requires stable predictions, thus 6 to 9 years of data was adopted to predict the next year’s incidence, respectively, to test the validity of the additive model of exponential smoothing method. From Table 1, it can be seen that the *R*^2^ of the model increased steadily from 0.927 to 0.949 with the increase of the data volume. From Fig. 5 and Table 2, it can be seen that all the actual values except for two in April and May 2017 fall in the confidence interval of predicted value.

**Table 1:** Sensitivity of the additive model

Fitted time	*R* ^2^	RMSE	MAPE	MaxAPE	MAE	MaxAE	Normalized BIC
Apr 2007 to Mar 2013	0.927	425.086	13.297	73.333	316.591	1317.517	12.283
Apr 2007 to Mar 2014	0.934	406.071	12.451	77.616	301.618	1375.224	12.171
Apr 2007 to Mar 2015	0.941	427.130	12.359	69.610	331.399	1222.761	12.257
Apr 2007 to Mar 2016	0.949	418.941	12.024	67.816	334.053	1243.163	12.206

*Note*: *RMSE* root mean square error, *MAPE* mean absolute percentage error, *MaxAPE* maximum absolute percentage error, *MAE* mean absolute error, *MaxAE* maximum absolute deviation, *Normalized BIC* normalized Bayesian information criterion

**Table 2:** Extrapolation prediction of human brucellosis cases in Mainland China

Time	Actual number of cases	Forecast number of cases	95% confidence interval
Apr 2017	4048	5213	[4398, 6028]
May 2017	4539	5968	[4970, 6966]
Jun 2017	5203	6246	[5094, 7399]
Jul 2017	4742	5942	[4654, 7230]
Aug 2017	4330	4915	[3504, 6326]
Sep 2017	2781	3367	[1843, 4891]
Oct 2017	1953	2421	[792, 4050]
Nov 2017	2427	2619	[892, 4347]
Dec 2017	2549	2727	[906, 4548]
Jan 2018	2202	2226	[316, 4136]
Feb 2018	1846	2515	[520, 4510]
Mar 2018	3755	4506	[2429, 6582]

## Discussion

In the present study, data accumulated in mainland China was used to provide an overview of the temporal distribution of human brucellosis cases and the feasibility of exponential smoothing method in the forecasting of incident human brucellosis cases was explored.

The study adopted the number of reported human brucellosis cases to present the temporal distribution in mainland China and the numbers that provided could give some hints to the allocation of brucellosis-related healthcare resources. The clear seasonality of reported human brucellosis was found in the present study, indicating more contact between human and infected livestock from March to August which is the cultivation time in the northern agricultural areas and the lambing time in the pastoral areas in China, also the warmer temperature might be suitable for the zoonotic transmission of pathogens [17, 18]. Temperature, monthly hours of sunshine and rainfall have shown the highest probabilities of influencing brucellosis transmission in China according to the Granger causality tests [11].

Models dealing with time series data have been traditionally used in econometrics, and recently their use is increasing in medical fields under the background of increasing data quality of disease surveillance database [19, 20]. Generally, the time series of disease surveillance sourced data can be decomposed into four components: the trend, the cyclical, the seasonal (systematic, calendar related movements), and the irregular pattern. This study targeted the application of exponential smoothing method to predict human cases of brucellosis based on historical time series. For exponential smoothing method, also known as exponential weighted average method, the major advantage of this model is that it chooses the weighted average of each period as a decreasing exponential sequence, giving greater weight to the historical data closer to the forecast period. It might be suitable for the forecast of time-series data with trends and seasonality. During the extrapolation prediction, all the actual values except for two (April and May 2017) fell in the confidence interval of predicted value (Fig. 5). If in the real world an actual incident value exceeds the upper confidence limit of the forecast, it will alert us the high emerging risk of the outbreak or epidemic which is necessary to put more special effort into the coping strategy and solutions of brucellosis, not only in medical and veterinary fields but also in economic areas [21–24].

The limitations of this study should be acknowledged. First, as brucellosis is a zoonotic disease caused by *Brucella spp.* and the transmission routes to humans are direct with infected animals, consumption of meat and dairy products, or intentional or accidental aerosol exposure [25, 26]. Monthly data of the abovementioned factors could not be obtained in the present study; we could only collect these data on an annual basis. The risk of the disease is mainly affected by the amount of livestock herds [27–29]. We have found that the Pearson correlation coefficient between the annual number of human brucellosis cases and cattle slaughter amount, sheep slaughter amount, beef yield, mutton yield, and wool yield is 0.803, 0.781, 0.642, 0.708, and 0.564, respectively, indicating the existence of strong correlations. Second, the analysis and forecast were based on the data that were collected from a passive surveillance system of infectious diseases. During the study period, the modifications of the laboratory diagnostic test, the diagnosis criteria of cases, and the availability of health-care providers and hospital resources might have an impact on the data quality. Third, data about the pathogens and their variations were not available in the present study. Fourth, we are not able to utilize data at the individual province level. However, the monthly data that we used were the most nationally official data of human brucellosis in mainland China.

## Conclusions

Human brucellosis cases peaked during the months from March to August in mainland China, with clear seasonality. The exponential smoothing that based on additive model method could be effectively used in the time series analysis of human brucellosis in China. Control methods, such as vaccination, quarantine, elimination of infected animals and good hygiene within the production cycle should be strengthened with paying more attention to the seasonality. Further research is warranted to explore the drivers behind the seasonality.

## Abbreviations

BIC: Bayesian Information Criterion
CDC: Center for Disease Control and Prevention
MAE: Mean absolute error
MAPE: Mean absolute percentage error
MaxAE: Maximum absolute deviation
MaxAPE: Maximum absolute percentage error
NIDR: National noticeable infectious disease reporting system
RMSE: Root mean square error
STL: Seasonal-trend decomposition
